# Odontogenic Abscess-Related Emergency Hospital Admissions: A Retrospective Data Analysis of 120 Children and Young People Requiring Surgical Drainage

**DOI:** 10.1155/2018/3504727

**Published:** 2018-08-26

**Authors:** Christian Doll, Fabian Carl, Konrad Neumann, Jan Oliver Voss, Stefan Hartwig, Richard Waluga, Max Heiland, Jan-Dirk Raguse

**Affiliations:** ^1^Charité–Universitätsmedizin Berlin, Corporate Member of Freie Universität Berlin, Humboldt-Universität zu Berlin, and Berlin Institute of Health, Department of Oral and Maxillofacial Surgery, Germany; ^2^Charité–Universitätsmedizin Berlin, Corporate Member of Freie Universität Berlin, Humboldt-Universität zu Berlin, and Berlin Institute of Health, Department of Radiology, Germany; ^3^Charité–Universitätsmedizin Berlin, Corporate Member of Freie Universität Berlin, Humboldt-Universität zu Berlin, and Berlin Institute of Health, Institute of Biometry and Clinical Epidemiology, Germany; ^4^Department for Oral and Maxillofacial and Facial Plastic Surgery, Johannes Wesling Hospital Minden, University Hospital of the Ruhr University Bochum, Germany

## Abstract

**Introduction:**

Even today, despite medical progress and intensive health education, odontogenic infections leading to surgical intervention and hospitalization are common in children and young people. The aim of this study was to give a detailed overview of clinical and economic data on children and young people treated and hospitalized due to an odontogenic abscess at a tertiary university hospital.

**Methods:**

A single-center retrospective analysis of patients under the age of 18 years who were hospitalized and surgically treated under local or general anesthesia for an odontogenic abscess during a period of 24 months was performed.

**Results:**

A total of 120 patients (77 males; 43 females) within the observation period of 2 years were included. The mean age was 6.3 years (ranging from 1 to 17 years). The most frequent diagnosis was a canine fossa abscess (n = 52; 43.3%) and the left primary maxillary first molar could be identified as the most frequent source of infection. The average length of hospital stay was 1.82 days (ranging from 0 to 8 days). The duration was significantly correlated with the kind of abscess diagnosed (p < 0.001) and the duration of the surgical intervention in patients who were treated under general anesthesia (rho = 0.259, p = 0.005). A statistically significant relationship was observed between the kind of abscess and cost (p < 0.001).

**Conclusion:**

The length of hospital stay was significantly correlated with the kind of abscess diagnosed. The left primary maxillary first molar could be identified as the most frequent source of infection. A statistically significant relationship was observed between the kind of abscess and cost.

## 1. Introduction

An odontogenic infection arises from a tooth or its supporting structures. It commonly occurs secondary to dental caries, periodontal disease, or pericoronitis and is caused by different types of bacteria. Main symptoms include pain, swelling, and erythema. If the primary source of the infection is not eliminated, the process of inflammation can progress and may result in severe (local as well as systemic) complications [1].

Odontogenic infections in children are a frequent reason for seeking medical care in an emergency department [2–4]. Although severe clinical cases are relatively rare, life-threatening complications are described in children [5, 6].

The therapeutic approach depends on various factors including the source and severity of the infection. The primary source has to be eliminated [1]. In case of a (nonpreservable) dental focus, timely extraction has been shown to be beneficial in most situations [7]. While most children presenting at the emergency department with an odontogenic infection can be discharged with oral antibiotics and necessary interventions can be performed at a later stage [8], patients suffering from severe infections including strong swelling, cellulitis, or abscess formation need to be treated immediately. In the latter case, surgical incision and drainage under local or general anesthesia are necessary to remove accumulated pus. In many of these cases, administration of intravenous antibiotics is necessary.

The treatment of odontogenic infections has meanwhile become a significant economic burden to public healthcare facilities, especially in patients requiring hospitalization [9–11].

Studies have shown that rapid treatment has a significant positive influence on the length of the inpatient stay [12, 13]. In pediatric dental treatment in general, an increasing role of the use of sedation/general anesthesia can be observed [14]. Especially in younger children general anesthesia is often indispensable due to reduced compliance.

Numerous publications exist that deal with clinical data of odontogenic infections in adults requiring hospitalization [11, 15–17]. However, there are only a few focusing exclusively on children and young people [8, 12, 13, 18, 19]. These provide only limited clinical data on children surgically treated under general anesthesia. Therefore, we conducted a retrospective clinical data analysis of patients under 18 years who were hospitalized due to an odontogenic abscess and surgically treated under local or general anesthesia.

The purpose of this study was to give a detailed overview of clinical and economic data on children and young people treated under local or general anesthesia and hospitalized due to an odontogenic abscess at a tertiary university hospital.

## 2. Materials and Methods

### 2.1. Ethics Statement

The Ethics Committee of the Faculty of Medicine Charité, Medical University Berlin, approved this study.

### 2.2. Study Design

A single-center retrospective data analysis of all patients presenting at the emergency room of our Department during a period of 24 months (between 04/01/2013 and 03/31/2015) was performed. Of these, hospitalized patients under the age of 18 years with the following ICD-10 (International Classification of Diseases, German Modification) codes were included: K12.21/22 (submandibular abscess), K12.23 (buccal abscess), K12.28 (massetericomandibular abscess; floor of mouth abscess; paramandibular abscess; perimandibular abscess), K12.29 (oral abscess), K04.7 (canine fossa abscess; dental abscess), and K10.20/21 (abscess of the upper jaw). Only patients who had been surgically drained in local or general anesthesia due to an odontogenic abscess were enrolled. The exact abscess diagnosis/localization of the abscess was extracted from the operation report. Patients were excluded as study subjects if they had only received i.v. antibiotic without surgical drainage. Furthermore, patients who had left against medical advice after admission without surgical intervention were excluded. Patients with insufficient/contradictory data were not considered.

Electronic patients' records were reviewed retrospectively with regard to individual patients' characteristics, demographic/economic parameters, etiology, clinical symptoms and diagnostic tests at the time of presentation, treatment, microbiology, and clinical course during the inpatient stay. Teeth are numbered according to the FDI (Fédération Dentaire Internationale) scheme.

### 2.3. Statistical Analysis

The data were collected in Microsoft Excel (Version 15.33; Microsoft Corporation, Redmond, WA, USA) and analyzed using IBM SPSS Statistics 25 (IBM Corporation, Armonk, NY, USA). For descriptive statistics, we give for each group frequencies and percentages for categorical and means and standard deviations for metrical variables. Associations between categorical variables were described by cross-tabulation and tested by Pearson's chi-square test. Student's t-test and analysis of variance (ANOVA) were used to compare means in different groups of symmetrically distributed metrical variables without outliers. Since the distribution of the variable “length of stay” was skewed, all statistical comparisons were performed using Mann–Whitney U and Kruskal-Wallis tests, where appropriate. Correlation between two metrical variables was determined by Spearman's rank correlation coefficient. The level of significance was *α*=0.05. Due to the exploratory nature of the study no Bonferroni adjustment for multiple testing was applied.

## 3. Results

**Figure 1:**
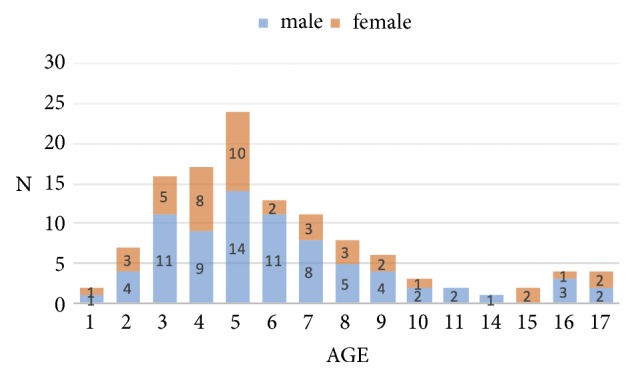
Age distribution in relation to gender.

A total of 120 patients (77 males; 43 females) accounting for 120 inpatient cases meeting the inclusion criteria were identified and included in the present study. The mean age of the population was 6.3 years, ranging from 1 to 17 years (Figure 1).

Whereas a similar gender distribution can be observed for most ages, there are considerable differences at the age of 6, where males were more affected (5.5:1).

Nine patients (9/120; 7.5%) were referred from other hospitals to our emergency department for further treatment. A total of 35 patients (35/120; 29.2%) reported some form of antibiotic treatment before presentation. In 14 patients (14/120; 11.7%), previous trepanation/endodontic treatment was documented. One patient (1/120; 0.8%) presented after initial surgical incision/drainage (alio loco) with persisting symptoms.

In 21 patients (21/120; 17.5%), preexisting conditions were documented. Of these, severe (chronic) disease was rare. Four patients had a history of heart disease. In two patients, a development disorder/mental retardation was known. One patient suffered from thalassemia major. Most of the children had no medical comorbidities. In three patients (3/120; 2.5%), a penicillin allergy was documented.

Most of the patients were of German citizenship (88/120; 73.3%). The most frequent foreign nationalities were Serbian (4/120; 3.3%) and Romanian (3/120; 2.5%). The citizenship of 12 children (12/120; 10.0%) was unknown.

The mean number of patients under the age of 18 per month who were admitted due to an odontogenic abscess and had surgical intervention under general or local anesthesia was 5.0. The highest incidence within the 2-year observation period was observed in summer (July; n = 20; 16.7%) and the lowest incidence in spring (April; n = 1; 0.8%). The majority of the patients presented in the first half of the month (days 1-15: n = 72; days 16-31: n = 48). Most of the patients showed up after the weekend on a Monday (Table 1).

A total of 97 patients presented between 7 a.m. and 7 p.m. Twenty-three presented during “on-call service” at night. The distribution of the time of presentation is summarized in Table 2.

Dental pain, swelling, and erythema were identified to be frequent symptoms. Furthermore, the following symptoms could be observed: limited mouth opening (29/120; 24.2%), dysphagia (10/120; 8.3%), painful palpation of the medial angle of the eye (7/120; 5.8%), and dyspnea (1/120; 0.8%).

A total of 121 abscesses were diagnosed in 120 patients at the time of presentation. In one patient, a paramandibular abscess and an additional (commencing) submucous abscess of different localization were diagnosed. However, only the main diagnosis “paramandibular abscess” was considered for further analysis.

The most frequent diagnosis within the study population was canine fossa abscess (n = 52; 43.3%), followed by submucous (n = 28; 23.3%) and paramandibular (n = 21; 17.5%) abscess. The distribution of the diagnoses, their respective main symptoms, and inflammatory blood values (C-reactive protein (CRP)/leukocytes) at the time of presentation are summarized in Table 3.

**Figure 2:**
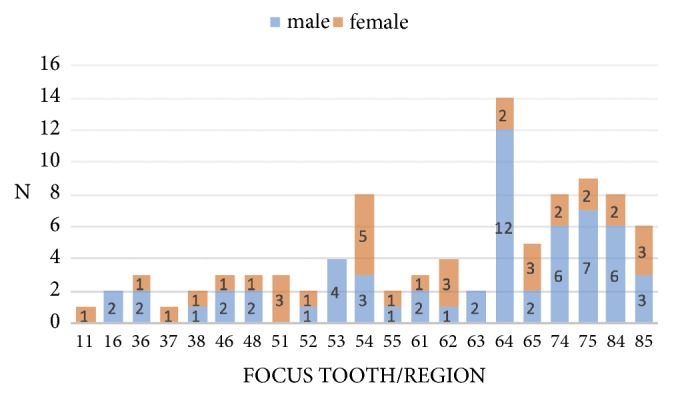
Focus teeth/regions in relation to gender.

Analysis of the cause of the odontogenic abscess revealed that most of the patients suffered from dental caries and/or apical periodontitis/pulpitis (106/120; 88.3%). Eight patients developed an abscess after tooth removal (8/120; 6.7%). The cause in six patients (6/120; 5.0%) was not clear. In 93 out of 120 patients (77.5%), a focus tooth/region of the infection could be identified (Figure 2). If there were two or more teeth as the potential focus in one patient, the data of this patient was not taken into account.

The tooth most frequently associated with an abscess was the primary maxillary first molar. In contrast to the lower jaw, infections on all primary teeth in the upper jaw played a causative role in abscess development. In comparison to all the other primary molars, the right primary maxillary second molar (tooth 55; FDI) was a relatively rare cause of an abscess. Distribution of the causative tooth/focus region according to the specific abscess diagnosis is presented in Table 4.

In 120 patients, a total of 120 primary surgical interventions (incision and drainage) under local (n = 4; 3.3%) or general anesthesia (n = 116; 96.7%) were performed.

The average period between registration at the emergency department and time of incision in the operating room in patients who were treated under general anesthesia was 6:28 hours, ranging from 46:12 minutes to 37:49 hours.

The mean duration of surgery was 20:10 minutes, ranging from 5:02 to 56:32 minutes. In three patients (n = 3; 2.5%), revision surgery had to be performed due to persistent abscess-related symptoms. During all surgical interventions (primary/revision; local/general anesthesia) a total of 264 teeth were extracted.

The average duration of the inpatient stay was 1.82 ±1.188 days, ranging from 0 to 8 days. One patient left at the day of hospitalization after the surgical intervention against medical advice (length of stay: 0 days). The characteristic features of the inpatient stay and the (primary) surgical procedures are summarized in Table 5.

The results of the statistical analysis are summarized in Table 6. A statistically significant relationship was not observed between length of inpatient stay and gender (p = 0.479; Mann–Whitney U test) or previous antibiotic treatment (p = 0.880; Mann–Whitney U test). However, the duration of the stay was significantly correlated with the duration of the surgical intervention in patients who were treated under general anesthesia (rho = 0.259, p = 0.005; Spearman correlation coefficient) and the kind of abscess diagnosed (p < 0.001; Kruskal-Wallis test). Patients suffering from a perimandibular or floor of mouth abscess were hospitalized more than twice as long as patients with a submucous/canine fossa abscess. A statistically significant relationship was observed between length of inpatient stay and age groups according to the dentition periods (p < 0.001; Kruskal-Wallis test). Patients between 14 and 17 years were hospitalized more than twice as long than patients under 6 years. A significant difference (p < 0.001; Mann–Whitney U test) was observed between the length of stay of patients who suffered from a primary focus tooth/region and a secondary focus tooth/region and patients in whom a focus tooth/region could not be identified.

A total of 45 surgical procedures (45/116; 38.8%) under general anesthesia were performed between 7 a.m. and 7 p.m. The majority of the surgical procedures (71/116; 61.2%) occurred between 7 p.m. and 7 a.m. (“on-call service”).

Pus swabs were sent for microbiological analysis (and antibiotic susceptibility) in 53 patients (53/120; 44.2%).* Streptococcus anginosus* and* Streptococcus mitis/oralis* were among the most frequent organisms found.

The majority of patients (114/120; 95.0%) had statutory insurance. The costs of two patients (2/120; 1.7%) were (directly) covered by the government (Regional Office for Health and Social Affairs Berlin; LAGeSo Berlin). Three patients (3/120; 2.5%) had private insurance. One patient had no form of insurance.

Economic data for 45 (of 48) cases for the 2014 calendar year were available for this study (Table 7). Of these cases, a total of 84.855 Euros was covered by reimbursements from insurances. In contrast, the charge (internal cost allocation) incurred by other departments (mainly anesthesiology) was 71.655 Euros. A statistically significant relationship was observed between the kind of abscess and cost (p < 0.001; Kruskal-Wallis test).

## 4. Discussion

Only a few studies exist that describe odontogenic infections in pediatric patients requiring hospitalization and these provide only limited clinical data on patients surgically drained in general anesthesia [8, 12, 13, 18, 19]. Therefore, we conducted this study, which is, to the best of our knowledge, one of the largest studies of this kind.

The mean age of the study population was 6.3 years (ranging from 1 to 17 years) and a higher incidence was observed in males (males: 64.2%; females: 35.8%; ratio: 1.79:1). The comparability of these parameters with other study evaluating pediatric patients requiring hospitalization due to an odontogenic infection appears difficult because of different age groups (between < 17 years and < 20 years) included [13, 18]. The mean age in a study by Kara et al. evaluating patients under 18 years was higher [12].

While three studies reported an equal gender distribution [13, 18, 20], our results are similar to those of Kara et al. where a male dominance (1.4:1) could be seen [12].

Most of the children evaluated in the present study had no (known) medical comorbidities. In 21 patients, preexisting conditions were documented. Of these, severe (chronic) disease was rare. These results are in line with the results found by Michael et al. evaluating in- and outpatient cases of odontogenic swelling in children at an emergency department [8]. In that study, the majority of the patients (93%) were “fit and healthy”.

In the present study, most of the children were of German citizenship (88/120; 73.3%). The hospital where the study was conducted is located in the Wedding district in Berlin. According to the report in June 2015 (A I 5 – hj 1 / 15) of the Statistical Office for Berlin-Brandenburg (2016), which is a central service provider for statistics in Berlin, about 30% of the people in this district are foreign citizens (Berlin in total: 15%). The present study therefore has a similar proportion of foreigners.

Most of the patients presented at the emergency department in summer (July; n = 20; 16.7%). Further numbers of presentations for the respective month were January (n = 9), February (n = 14), March (n = 6), April (n = 1), May (n = 10), June (n = 14), August (n = 9), September (n = 11), October (n = 8), November (n = 7), and December (n = 11). In contrast, Kara et al. observed that January was the peak month for admission of pediatric patients suffering from odontogenic infections [12]. Unkel et al. observed that admissions for odontogenic cellulitis peaked in spring [20]. Lin et al. reported that the peak occurrence month for pediatric odontogenic cellulitis was February for in- and outpatients [19]. In this study, most of the patients presented during the warm (June/July/August: n = 43) or cold season (December/January/February: n = 34) in comparison to the transition periods of spring (March/April/May: n = 17) and autumn (September/October/November: n = 26). The exact reason for this cannot be concluded from our data. However, it has been shown that ambient temperature has a considerable impact on the oral temperature [21, 22] and that even small changes of the temperature can influence bacterial growth within the oral microbial flora [23]. It might be possible that the exposure to warm/cold outdoor temperatures might influence the growth of (specific) bacteria which promote the development of dental abscesses.

Most of the patients were referred directly before (Friday; n = 20; 16.7%) and after (Monday; n = 23; 19.2%) the weekend, which in our opinion could have been due to the opening hours of the doctors in charge. The majority of the patients (97/120; 80.1%) presented between 7 a.m. and 7 p.m. However, most of the surgical procedures (71/116; 61.2%) in general surgery were performed between 7 p.m. and 7 a.m. (“on-call service”). This fact has to be considered for human resource and operation theatre planning. Since operative room time during night is more expensive, this represents an extra financial burden for hospitals. Other studies evaluating odontogenic infections in pediatric patients who require hospitalization do not provide data regarding time of presentation and time of surgery [8, 12, 13, 18, 19].

The average duration of the inpatient stay was 1.82 ±1.188 days, ranging from 0 to 8 days. The duration of the stay was significantly correlated with the kind of abscess diagnosed (p < 0.001). As expected, the length of stay of patients who presented with a (large) space abscess was longer than of patients who suffered from a submucous abscess. Furthermore, the duration of the stay was significantly correlated with the duration of the surgical intervention in patients who were treated under general anesthesia. However, this correlation is weak and a larger sample size is required to verify this result.

In contrast to the study of Kara et al. [12], a statistically significant relationship was observed between length of inpatient stay and different age groups (p < 0.001). The mean duration was longer in older patients. This is due to the fact that older children in this study suffer more frequently from (large) space abscesses.

In comparison to most of the other studies evaluating pediatric patients requiring hospitalization due to an odontogenic infection (mean length ranging from 5.03 to 5.86 days) [12, 18, 19], the mean duration of the hospital stay in the present study was considerably shorter. However, comparison of the length of stay between this study and other studies [12, 18, 19] seems difficult. In contrast to the present study, other authors classified the infections as upper face or lower face according to their anatomic location modified from Dodson's definition [24]. The exact proportion of (severe) space infections remains unclear. Nonetheless, our results are comparable with the mean length of stay found by Thikkurissy et al. [13]. As described by these authors, we also conclude that the early elimination of the source of infection (and therefore definitive treatment) is one of the main reasons for the very low average duration of the hospital stay. Also, Kara et al. found that the length of stay was significantly shorter in patients who had a tooth extraction within 48 hours in comparison to patients who had been treated at 48 hours or later [12].

In the present study, tooth 64 could be identified as the most frequent source of infection. The primary first molar was responsible for the most abscesses in the upper and lower jaw, as already observed by other authors [12, 18].

As mentioned, economic data for 45 cases for the 2014 calendar year was available for this study. Of these cases, a total of 84.855 Euros was covered by reimbursements from insurances. In contrast, the charges (internal cost allocation) incurred by other departments (mainly anesthesiology) were 71.655 Euros. Thus, there was a profit of about 13.200 Euros which is nowhere near enough to cover the further expenses to treat these patients. Therefore, this kind of disease is an economic burden for the respective department.

The present study provides detailed clinical and economic data on 120 patients at a large tertiary university hospital; however, there are a few limitations. Although we evaluated a comparably large amount of patients, the analysis of more patients in a prospective approach is necessary to confirm the results of this study.

## 5. Conclusion

The length of hospital stay was significantly correlated with the kind of abscess diagnosed. The left primary maxillary first molar could be identified as the most frequent source of infection. A statistically significant relationship was observed between the kind of abscess and cost.

## Figures and Tables

**Table 1:** Weekday of presentation at the emergency department.

**Weekday**	**N (%)**
Monday	23 (19.2%)
Tuesday	19 (15.8%)
Wednesday	13 (10.8%)
Thursday	15 (12.5%)
Friday	20 (16.7%)
Saturday	12 (10.0%)
Sunday	18 (15.0%)

**Table 2:** Time (frame) of presentation at the emergency department.

**Time**	**N (%)**
6 a.m. – 11:59 a.m.	33 (27.5%)
12 p.m. – 4:59 p.m.	51 (42.5%)
5 p.m. – 11:59 p.m.	31 (25.8%)
12 a.m. – 5:59 a.m.	5 (4.2%)

**Table 3:** Distribution of patients according to diagnoses as per the mean age, side, main symptoms, and values of CRP and leukocytes.

**Abscess diagnosis**	**N (%)**	**Age in years** (Mean ± SD)	**Localisation** **(Right/Left)**	**Symptoms**				**Inflammatory values**	
				**Dysphagia**	**Dyspnea**	**LMO**	**PP**	**CRP** **mg/l** (median [IQR])	**Leukocytes** **/ nl** (mean ± SD)
Submucous	28 (23.3%)	5.0 ± 2.27	11/18*∗*	1 (3.6%)	0 (0%)	0 (0%)	0 (0%)	16.55 [48.05] (n = 4)	10.1 ± 2.21 (n = 4)
Canine fossa	52 (43.3%)	5.7 ± 3.51	20/32	1 (1.9%)	0 (0%)	4 (7.7%)	7 (13.5%)	23.9 [49.1] (n = 7)	11.4 ± 3.93 (n = 8)
Paramandibular	21 (17.5%)	5.9 ± 3.35	10/11	1 (4.8%)	0 (0%)	10 (47.6%)	0 (0%)	18.3 (n = 3)	10.9 ± 1.93 (n = 3)
Perimandibular	14 (11.7%)	9.9 ± 4.38	6/8	6 (42.9%)	0 (0%)	10 (71.4%)	0 (0%)	55.4 [81.25] (n = 5)	15.9 ± 6.33 (n = 7)
Floor of mouth	3 (2.5%)	8.0 ± 1.00	3/1*∗*	1 (33.3%)	1 (33.3%)	3 (100%)	0 (0%)	48.6 (n = 1)	15.3 (n = 1)
Massetericomandibular	2 (1.7%)	16.0 ± 0.00	2/0	0 (0%)	0 (0%)	2 (100%)	0 (0%)	38.2 (n = 2)	13.9 ± 3.31 (n = 2)

*∗* one abscess occurred on both sides.

LMO: limited mouth opening.

PP: painful palpation of the medial angle of the eye.

**Table 4:** Distribution of teeth/regions responsible for the specific abscess.

**Abscess diagnosis**	**Causative tooth/focus region**																				
	**11**	**16**	**36**	**37**	**38**	**46**	**48**	**51**	**52**	**53**	**54**	**55**	**61**	**62**	**63**	**64**	**65**	**74**	**75**	**84**	**85**
Submucous						1		2			2			1		2		5	3	3	2
Canine fossa	1	2						1	2	4	6	2	3	3	2	12	5				
Paramandibular			1		1		1											3	4	5	3
Perimandibular			2	1	1	1	1												2		1
Floor of mouth						1															
Massetericomandibular							1														

**Table 5:** Distribution of patients according to diagnoses as per the duration of inpatient stay, form of anesthesia, duration of intervention, surgical approach, and type of drain.

**Abscess diagnosis**	**N (%)**	**Duration of inpatient stay (days)** (mean ± SD)	**Form of anesthesia**		**Duration of intervention in GA (min)** (mean ± SD)	**Approach **		**Type of drain**		
			**LA**	**GA**		**IO**	**EO**	**EF/PR**	**JF**	**None**
Submucous	28 (23.3%)	1.11 ± 0.497	0	28	15:22 ± 5:33	28	0	5	11	12
Canine fossa	52 (43.3%)	1.56 ± 0.639	2	50	20:52 ± 12:36	52	0	10	30	12
Paramandibular	21 (17.5%)	1.81 ± 1.031	1	20	18:19 ± 7:31	21	0	3	14	4
Perimandibular	14 (11.7%)	3.21 ± 1.188	1	13	29:00 ± 8:49	3	11	11*∗*	2*∗*	0*∗*
Floor of mouth	3 (2.5%)	5.33 ± 2.517	0	3	21:18 ± 1:32	1	2	3	0	0
Massetericomandibular	2 (1.7%)	3.50 ± 0.707	0	2	28:34 ± 2.02	1*∗∗*	2*∗∗*	2	0	0

EF/PR: Easy-Flow/Penrose; JF: Jodoform gauze; LA: local anesthesia; GA: general anesthesia; EO: extraoral; IO: intraoral; *∗* type of drain could not be assessed precisely in one patient; *∗∗* intra- and extraoral drainage in one patient.

**Table 6:** Statistical results.

**Comparison**	**Parameter**	**N**	**LOS** (mean ± SD)	
Gender vs. LOS	Male	77	1.82 ± 1.167	p = 0.479
	Female	43	1.81 ± 1.240	

Previous antibiotic treatment vs. LOS	No	85	1.81 ± 1.190	p = 0.880
	Yes	35	1.83 ± 1.200	

Abscess diagnosis vs. LOS	Submucous	28	1.11 ± 0.497	p < 0.001
	Canine fossa	52	1.56 ± 0.639	
	Paramandibular	21	1.81 ± 1.031	
	Perimandibular	14	3.21 ± 1.188	
	Floor of mouth	3	5.33 ± 2.517	
	Massetericomandibular	2	3.50 ± 0.707	

Focus tooth/region vs. LOS	Primary	78	1.50 ± 0.752	p < 0.001
	Secondary	15	3.13 ± 1.408	
	No focus identified	27	2.00 ± 1.544	

Age groups vs. LOS	< 6	66	1.47 ± 0.749	p < 0.001
	6-13	43	2.02 ± 1.504	
	14-17	11	3.09 ± 0.944	

**LOS**: length of stay.

**Table 7:** Economic data with costs and reimbursements of 45 cases.

**Abscess diagnosis**	**N**	**Cost in Euros** (mean ± SD)	**Reimbursement in Euros** (mean ± SD)
Submucous	11	1236.15 ± 285.253	1333.38 ± 183.053
Canine fossa	16	1463.92 ± 420.243	1620.64 ± 337.865
Paramandibular	7	1356.80 ± 498.422	1460.26 ± 381.256
Perimandibular	7	2296.51 ± 656.973	3038.09 ± 846.460
Floor of mouth	2	2587.18 ± 769.280	3192.18 ± 0.000
Massetericomandibular	2	1943.33 ± 81.423	3192.18 ± 0.000

## Data Availability

The data used to support the findings of this study are included within the article.
